# A qualitative investigation of uninsured patient and primary care provider perspectives on specialty care eConsults

**DOI:** 10.1186/s12913-023-10086-6

**Published:** 2023-10-20

**Authors:** Lauren Bifulco, Lynsey Grzejszczak, Idiana Velez, Tracy Angelocci, Daren Anderson

**Affiliations:** 1 Weitzman Institute, 19 Grand Street, Middletown, CT USA; 2 Lone Star Circle of Care, 205 East University, Suite 100, Georgetown, TX USA

**Keywords:** Access to Care, Health Disparities, Patient perspectives, Provider perspectives, Primary care, eConsults

## Abstract

**Background:**

Uninsured and underinsured patients face specialty care access disparities that prevent them from obtaining the care they need and negatively impact their health and well-being. We aimed to understand how making specialty care electronic consultations (eConsults) available at a multi-site Federally Qualified Health Center (FQHC) in central Texas affected uninsured patients’ care-seeking experiences and impacted their ability to receive the needed care.

**Methods:**

We used concepts from Ecological Systems Theory to examine individual, interpersonal, organization-level, social, and health policy environment factors that impacted patients’ access to specialty care and the use of eConsults. We conducted thematic analysis of semi-structured, qualitative interviews with patients about seeking specialty care while uninsured and with uninsured patients and FQHC PCPs about their experience using eConsults to obtain specialists’ recommendations.

**Results:**

Patients and PCPs identified out-of-pocket cost, stigma, a paucity of local specialists willing to see uninsured patients, time and difficulty associated with travel and transportation to specialty visits, and health policy limitations as barriers to obtaining specialty care. Benefits of using eConsults for uninsured patients included minimizing/avoiding financial stress, expanding access to care, expanding scope of primary care, and expediting access to specialists. Concerns about the model included patients’ limited understanding of eConsults, concern about cost, and worry whether eConsults could appropriately meet their specialty needs.

**Conclusions:**

Findings suggest that eConsults delivered in a primary care FQHC addressed uninsured patients’ specialty care access concerns. They helped to address financial and geographic barriers, provided time and cost savings to patients, expanded FQHC PCPs’ knowledge and care provision options, and allowed patients to receive more care in primary care.

**Supplementary Information:**

The online version contains supplementary material available at 10.1186/s12913-023-10086-6.

## Background

High-quality primary care is comprehensive, long-term, person-centered, and coordinated [1]. Primary care providers (PCPs) are the first point of contact for diagnosis and management of common conditions as well as de-facto care-coordination hubs for patients who need more complex care for specialized conditions [2]. Access to high quality primary care is an essential component of the health care system that improves health outcomes [3].

Federally Qualified Health Centers (FQHCs) provide high quality primary care to over 30 million patients across the U.S. regardless of income, employment status, residency status, health insurance coverage, or ability to pay for care [4]. Medicaid expansion has increased health insurance coverage in most states and improved access to health services including primary care. Many of the patients benefiting from Medicaid expansion receive care in FQHCs [5]. However, in the 12 states that have not expanded Medicaid, an estimated 31.6 million remain uninsured, with negative health outcomes such as an increase in burden of chronic diseases, as well as shorter life expectancy [6, 7]. There are approximately 5.4 million uninsured people in Texas, which has not expanded Medicaid; 771,000 of these uninsured adults fall into the Medicaid coverage gap [8]. Patients in non-expansion states such as Texas are more likely to report variations in ability to access medical care compared to their counterparts in expansion states [9]. The Kaiser Family Foundation reports that 13.5% of Texas adults report not seeing a doctor in the past 12 months due to the cost of care [10]. FQHCs and other safety net practices provide a partial solution to this problem by providing access to primary care. However, patients needing specialty care face limited options. Studies show that nearly one in three patients seen in primary care are referred to a specialist each year [11–13]. Specialty care is less likely to be available in underserved communities and more likely to be financially out of reach for many patients [14–16].

Electronic consultations (eConsults) are an emerging telehealth tool to help improve access to specialty care. eConsults are asynchronous exchanges of clinical information between PCPs and specialists about specific patients [17]. PCPs’ consult questions are transmitted to a specialist in a secure, HIPAA-compliant manner along with relevant supporting documentation. The specialist reviews the information and provides a consult note with suggestions for how to manage the case. Sometimes the eConsult will provide the necessary input and obviate the need for a face-to-face referral. Other times, a face-to-face visit may still be needed, but advice on appropriate next steps to optimize the workup and manage the case in the interim is provided. As such, eConsults improve access by reducing demand for face-to-face referrals, and optimizing care for those that need to be seen in person. Studies of eConsults have shown that they improve access to care, [18–20] reduce emergency room use, [18] lower the cost of care, [21–24] and reduce wait times for specialty care visits [25–27].

While these studies have demonstrated improved healthcare efficiency and cost savings, less is known about views and impacts of the service on patients and their PCPs [28]. Limited research suggests that patients and PCPs have a positive opinion of eConsults [17, 29, 30]. while few studies have explored uninsured patients’ perceptions about specialty care and the referral process.

We describe a qualitative investigation of PCPs’ and patients’ experience seeking specialty care and using eConsults at a large, multi-site Federally Qualified Health Center (FQHC) in Central Texas. We address the following research questions: (1) How did specialty eConsults affect uninsured patients’ experience obtaining care? (2) What did patients and primary care providers perceive as the benefits and drawbacks of using eConsults to enhance specialty care availability?

## Methods

### Design

This study was a cross-sectional qualitative evaluation of PCP-users of eConsults and patients who received an eConsult in response to a specialty consult request. We followed the Standards for Reporting Qualitative Research (SRQR) reporting guidelines for qualitative studies. The Community Health Center, Inc. Institutional Review Board approved the study protocol (#1189, 9/23/2021), and staff and patients provided verbal informed consent to participate.

### Setting

The study was conducted in a large FQHC with multiple practice sites located across central Texas. Approximately 20–30% of the FQHC’s patient population is uninsured and approximately 2/3 live in census-designated rural areas. The eConsult process was implemented across all of the FQHC’s sites in August of 2018 and expanded over time to include 15 adult and pediatric specialties: Allergy, Cardiology, Dermatology, Endocrinology, Gastroenterology, Hematology, Nephrology, Neurology, Orthopedics, Otolaryngology, Pain Medicine, Pulmonology, Rheumatology, and Urology. Beginning in September 2021 PCPs were able to request videoconference telehealth visits for uninsured patients who needed care for Cardiology, Endocrinology, Gastroenterology, and Rheumatology and for whom an eConsult was deemed insufficient. To support uptake and minimize added burden on primary care providers, the eConsult submission process was designed to follow the same steps as were used to request in person specialty referrals. Details of the process have been previously described [26]. Briefly, a dedicated eConsult referral coordinator identified referral requests for patients that were uninsured, gathered relevant materials from the electronic health record and submitted the eConsult to the specialty network using a secure, HIPAA-compliant data transmission process. Referrals that indicated a specific need for a face-to-face visit such as those requiring a procedure were scheduled by the coordinator and not sent as eConsults. All costs related to eConsults were covered by a multi-year grant intended to expand capacity of safety-net practices to manage specialty care for vulnerable populations in central Texas.

### Timeline

Qualitative interviews were conducted between February and August 2022 as part of a larger study involving chart review of 100 randomly selected eConsult requests submitted between June 1, 2020 and May 31, 2021. 

### Participants

**Fig. 1:**
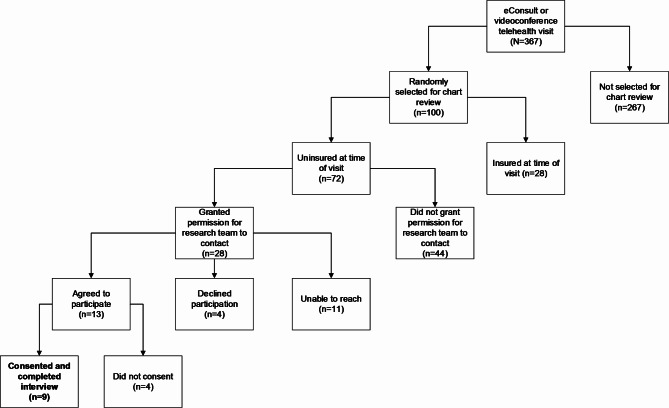
Patient Interview Recruitment

Patients were eligible for interviews if they were ≥ 18 years old, uninsured, English-speaking or Spanish-speaking, and had received an eConsult or PCP-requested videoconference telehealth visit with a specialist between June 1, 2020 and May 1, 2022. Twenty-eight patients agreed to be contacted by a member of the research team and nine patients consented to participate and completed 30-minute semi-structured telephone interviews. (Fig. 1). All PCPs who requested specialty eConsults for one or more patients during this timeframe (n = 25) were eligible for qualitative interviews. Nine PCPs consented to participation and completed 30-minute semi-structured videoconference interviews.

### Measures

#### Demographics

Patient demographic information included age, sex, race, Hispanic/Latino ethnicity, preferred language, and insurance status. PCP information included age, sex, race, Hispanic/Latino ethnicity, job title, time employed by the health center, and use of eConsults by frequency and specialty.

#### Interview guides

Ecological models are widely used in health-related research to better understand the intersecting factors that influence experiences and behavior [31, 32]. We employed concepts from Ecological Systems Theory [33] to develop semi-structured interview guides for patients and primary care providers. Questions were intended to elicit responses about individual experience, interpersonal interactions (e.g. the patient-provider relationship), and the impact of organization-level and social rules, policies, and environmental circumstances on patients’ experience obtaining specialty care. Patients were asked about their experience receiving specialty care while uninsured while PCPs were asked about their experience providing primary care to uninsured patients in need of specialty consultation. We asked both patients and PCPs for their perspectives on the organization-level, social, and health policy environment factors that might act as barriers or facilitators to specialty care for uninsured patients. Please see Appendix 1 for interview guides.

#### Analytic methods

We followed the Framework analysis process [34] to conduct thematic analysis of transcripts of patient and PCP interviews. Four researchers completed an analysis that entailed familiarization with the data through initial review of transcripts, generating initial themes, refining initial themes using a matrix to compare within cases (rows) and across cases (columns), and generating a report of individual, interpersonal, organizational, and systems and policy-level factors that contributed to patient and PCP perceptions. Analyses were conducted using Microsoft Excel and NVivo version 12 (QSR International, Sydney, QLD, Australia).

## Results

### Participant characteristics

The majority of interviewed patients were female (n = 7; 77.8%), aged 50–59 (n = 4; 44%; Mean = 46.5, SD = 7.7) and 44% (n = 4) identified as Hispanic or Latino. All patients were uninsured at the time of the eConsult. Interviewed PCPs were mostly female (n = 7; 77.8%), averaged 44.8 years old (SD = 12.9) and had worked at the FQHC for a mean of 6.4 years (SD = 4.2). (Table 1). The nine interviewed PCPs submitted 189 eConsults (Mean = 21.1, Range 3–40) in 15 different specialties (Mean = 7.1, Range 2–10) during the study period. The most common specialties were Gastroenterology (n = 53, 28.0%), Rheumatology (n = 29, 15.3%), and Hematology (n = 21, 11.1%). (Table 2). All interviewed PCPs used more than one eConsult specialty.

**Table 1:** Demographic Characteristics of Interviewed Patients (n = 9) and PCPs (n = 9)

Characteristic	(Patients, n = 9)	(PCPs, n = 9)
	N(%) or M(SD)	N(%) or M(SD)
**Age (M, SD)**	46.5 (7.7)	44.8 (12.9)
20–29	0 (0.0%)	1 (11.1%)
30–39	2 (22.2%)	2 (22.2%)
40–49	3 (33.3%)	2 (22.2%)
50–59	4 (44.4%)	2 (22.2%)
≥60	0 (0.0%)	2 (22.2%)
**Gender**		
Female	7 (77.8%)	7 (77.8%)
Male	2 (22.2%)	2 (22.2%)
**Race**		
American Indian or Alaska Native	1 (11.1%)	0 (0.0%)
Asian or Pacific Islander	1 (11.1%)	3 (33.3%)
Black or African American	0 (0.0%)	3 (33.3%)
White	6 (66.7%)	2 (22.2%)
Other/Unspecified	1 (0.0%)	1 (11.1%)
**Ethnicity**		
Hispanic/Latino	4 (44.4%)	0 (0.0%)
Not Hispanic/Latino	3 (33.3%)	9 (100.0%)
Other/Unspecified	2 (22.2%)	0 (0.0%)
**Insurance**		
Uninsured	9 (100%)	
**Preferred Language**		
English	5 (55.5%)	
Spanish	4 (44.4%)	
**Health Center Role**		
Nurse Practitioner		4 (44.4%)
Physician		5 (55.6%)
**Years at Health Center (M, SD)**		6.4 (4.2)
**# eConsult Specialties Used (M, SD)**		7.1 (2.8)

**Table 2:** eConsults Used by 9 Interviewed PCPs, by Specialty (n = 189)

Specialty	eConsults (n, %)
Gastroenterology	53 (28.0)
Rheumatology	29 (15.3)
Hematology	21 (11.1)
Neurology	17 (9.0)
Urology	14 (7.4)
Cardiology	13 (6.9)
Endocrinology	12 (6.3)
Dermatology	10 (5.3)
Nephrology	6 (3.2)
ENT	5 (2.6)
Orthopedics	4 (2.1)
Pain Medicine	2 (1.1)
Allergy	1 (0.5)
Infectious Disease	1 (0.5)
Pulmonology	1 (0.5)
	189 (100.0) (m = 21.1, SD = 12.3)

### Interview themes

Patients and PCPs provided substantial insights regarding the experience of seeking specialty care while uninsured. In addition, they offered insights as to the benefits and potential concerns with the eConsult model. (Table 3). While the focus of the study and the interview questions was on uninsured patients, we noted that at times, PCPs spoke about their use of eConsults more broadly, including comments regarding their use for patients with insurance who had complex conditions and access limitations.

**Table 3:** Interview Themes

Theme	Subtheme
Seeking Specialty Care While Uninsured	Feeling Stigmatized When Seeking Care
	Cost as a Barrier
Benefits of eConsults for Uninsured Patients	Minimizing/Avoiding Financial Stress
	Expanding Access to Care
	Expanding Scope of Primary Care
	Expediting Specialty Care When Needed
Concerns and Limitations of Using eConsults with Uninsured Patients	Patients’ Limited Understanding of eConsults
	Patient Concern about Cost
	Preference for In-Person Specialty Care
	Limitations of the Model
	Workflow Issues

### Seeking specialty care while uninsured

Several patients expressed that they felt marginalized or disregarded when trying to obtain care from specialists, and some voiced concern that specialty providers were not taking their needs seriously. A patient described having to choose between good quality care and affordable care: “I had to either go put up with doctors who wouldn’t listen to me or pay out of pocket for doctors who was (sic.) going to listen to me. And I had to choose the less expensive.” **(Patient #2).**

Uninsured patients who attempted to visit a specialist despite the out-of-pocket cost faced challenges scheduling appointments and expressed dissatisfaction with the care they received. A patient recalled: “I told them I didn’t have insurance. The person that called me goes, ‘Oh my God I wish they told me that! Well if you can come up with $200 she’ll talk to you for 10 minutes.’ And I thought, ‘well what is that going to do for me?’” **(Patient #8).** The ordeal of seeking care made one patient question the commitment of physicians to care for patients in need. They explained, “It was a little depressing… I had thought that doctors were there to help and make people’s lives better instead of worrying about money in their pocket. But I have found out through the years that that doesn’t stand always.” **(Patient #2)**.

PCPs understood that the cost of obtaining insurance was prohibitive for most patients. One observed, “Most of my patients without insurance have no insurance because they can’t afford it in the first place.” **(PCP #3).** Another described the broad impact of statewide health policy decisions on patients’ access to care: “The [lack of] Affordable Healthcare [Act] acceptance in Texas makes commercial insurance not so much subsidized for patients, since they’re not able to get those kind of insurances. So if they’re not able to get it they can’t see specialists.” **(PCP #6).**

### Benefits of eConsults for uninsured patients

#### Minimizing/avoiding financial stress

Patients pointed to out-of-pocket cost as the major barrier to specialty care, as many specialist offices required office visit payment to be provided in advance. A patient who had been unable to see a Rheumatologist stated, “I know that I need to see a specialist, but it’s not within my economic means to be able to contact one.”**(Patient #6).** Patients who realized that eConsults could reduce or eliminate the need for some face-to-face specialty visits found them beneficial. A patient stated, “It’s a very good option, because you don’t have to go somewhere else to have those types of tests done in another office… for me, the benefit would be that I don’t have to cover an extra consultation.” **(Patient #7).** A PCP noted the emotional and financial stress patients experience when seeking specialty care: “Patients just want to be reaffirmed sometimes…. And that’s what an eConsult can be very important for. Especially if the patients don’t have insurance, and they have difficulty getting where they want to go, or they can’t afford co-pays to see a lot of different specialists.” **(PCP #1).**

#### Expanding access to care

PCPs recognized that their patients faced numerous intersecting barriers to accessing specialty care on their own. A PCP noted that specialty referrals for uninsured patients often never occur: “If they don’t have insurance, the only option sometimes they have actually is… self-referral. They’ll send them a list, and the patient has to schedule themselves, so it may never get done.” **(PCP #1).** A second PCP observed that some specialists were not prepared to care for uninsured patients, explaining, “Those that are uninsured and have no payment scheme might not even be allowed to even make an appointment, because some of the specialists won’t even take payment plans or cash.” **(PCP #9).** PCPs also commented on general specialist shortages, which made it even more difficult for uninsured patients to obtain an appointment: “Endo[crinology] is a little harder to get a face-to-face sometimes - they have a lot of hoops to jump through to get to see an Endo[crinologist]…We had a shortage where at one time it could take a year to see a Rheumatologist, and still have to do a workup. **(PCP #5).**

eConsults also helped address geographic access challenges for certain patients. A PCP explained, “[the health center provides care] over six counties and so the nearest specialist might be 30, 40 [minutes], an hour away. So if you’re in Taylor, and even Round Rock takes 20, 30 to 40 minutes to get to. But [if] you have to go to Cedar Park for your specialist that’s literally probably an hour and a half drive, and not everybody has the time or the money.” **(PCP #9).** Another described obtaining an eConsult for a patient who had difficulty traveling even short distances for care: “I have a patient [and the patient’s partner], she doesn’t see very well, he doesn’t drive very well, he doesn’t really speak English well. She can, but she also can’t operate as well, so sometimes that issue, the drivability, feasibility those kind of things would be easier for an eConsult, if it’s something that may not really need the in-clinic per se.” **(PCP #1).**

#### Expanding scope of primary care

Patients acknowledged that eConsults were useful when their specialty care options were limited, and some expressed a preference for their PCP to direct their care. A patient described their strong relationship with their PCP and preference for the PCP to request an eConsult versus a specialty referral: “I trust her with everything that I am… I know that she’s not going to allow anything bad to happen to me… So I’d rather her be the one to tell me what I need to do.” **(Patient #8).** Another patient who preferred eConsults to specialty visits felt that their PCP had done more to address their concerns than a specialist they had previously seen: “She tried to give me some medicines and tests, and… find a solution for my pain. She never asked about insurance. The best was working with [PCP].” **(Patient #9).**

PCPs appreciated that eConsults helped them manage medically complex patients in primary care. A PCP described using eConsults to make small but important changes to patients’ medications – a treatment plan that utilized a specialist’s expertise, but could be implemented in primary care: “If I can just get a Cardiologist to look at a patient – this is what his blood pressure has been looking like, and these are the medications that he’s on, and these are his labs. You know, they’ll go in and rearrange medications. 'Oh, yeah, turn this one down, turn this one up, stop this one, start this one.' And that changes everything for the patient, and it’s not something that I would have identified.” **(PCP #2).** Another PCP described the general utility of eConsults for medically complex patients with needs beyond the typical scope of primary care: “It helps a lot when there’s more things going on, that we might not have a good answer for, or just don’t have that knowledge base for. So it helps a lot with trying to do some more in-depth work-up for patients who don’t have the ability to afford a specialist.” **(PCP #5).**

PCPs recognized the educational benefits of eConsults and how they helped expand their scope of practice: “I learn from recommendations. I actually have somewhere where I write stuff down based on patient profile, so I can use that knowledge…We can always send an eConsult… [but] if I have a similar patient with a similar profile I’m gonna start with the treatment knowledge that I have so far. Then, if I need help, then I reach out. **(PCP #7).** A nurse practitioner described how eConsults enabled them to expand their scope of care, stating, “[eConsults] help me to work at the top of my license… I can order procedures, and I can order medications that are not something that a family nurse practitioner would typically order.” **(PCP #2).**

PCPs also described using eConsults to build or reinforce specialty knowledge. One stated, “[eConsults] help educate our providers because they are able to then go in and go, ‘Oh, yeah, the last time I did one of these [for] a patient like this, and I did eConsult I had to do A, B, and C, so let me do A, B, and C and then send off the eConsult to see if I’m on the right path.’” **(PCP #10).** Another offered, “[eConsults are useful] to see if there’s more you need to do or if there’s something you’re missing…and give us some stuff that we can do to start working a patient up or give us a definitive answer [about] what we need to do next and a treatment plan.” **(PCP #9).**

#### Expediting specialty care when needed

PCPs had an overall positive opinion of eConsults as a service for immediate access and minimal waiting time. A frequent eConsult user stated, “[I request an eConsult] at least 15 to 20% of the time, because a lot of our population is uninsured.” **(PCP #7).** A PCP described patients’ appreciation that eConsults could expedite specialty care when it was truly needed: “Most of the time [patients are] grateful…If they need a follow up face-to-face visit, they understand that [submitting an eConsult] really cuts down on the waiting time, and it just strengthens the actual need for a face-to-face consult.” **(PCP #10).** Another discussed using an eConsult to help convince a patient to prioritize visiting a specialist: “If one of the specialists recommends an in-person visit, it is more likely to get them to go…it helps to encourage them to spend money they may not want to, and to go that route, and I tell them sometimes it is worth that.” **(PCP #2).**

### Concerns and limitations of using eConsults with uninsured patients

#### Patients’ limited understanding of eConsults

Although patients were aware that their PCPs sometimes conferred with colleagues about treatment recommendations, not all remembered their providers explaining what an eConsult was, and some did not realize that their PCP had obtained an eConsult to help with their treatment. Conversely, PCPs stated that they always explained what an eConsult was, and always told patients when they were requesting an eConsult. Some said that they also asked the patient to schedule a follow-up visit to review the recommendations together.

Most PCPs felt that patients generally understood eConsults, but some were not certain that patients would understand the eConsult process and its benefits. One stated, “It’s kind of a new concept to them …you tell them that you sent an eConsult in and they think they get a video visit with a specialist. And [I] tell them no, no, no, it’s just that they review the chart and give us feedback…I think at times patients think getting the eConsult is the same as a regular consult, and it’s not, obviously.” **(PCP #8).** Patients’ comments corroborated this PCP’s observation. One patient understood that their PCP had consulted with another doctor, but did not realize that the exchange had helped them avoid an unnecessary specialist visit: “I do remember that [PCP] talked to somebody else but the recommendation they gave was basically nothing. …Their recommendation was just to monitor and watch and that was it.” **(Patient #2).**

Some patients’ specific concerns reflected a lack of understanding about the role of eConsults and how they were used. One patient agreed that eConsults could be beneficial to PCPs and patients but expressed confusion about whether PCPs would then stop referring patients to specialists altogether, asking “How would that [an eConsult] work if its needing to be surgery?” (**Patient #2)**. Another patient recalled feeling uncertain about whether she still needed a specialist appointment, and subsequently learning that her PCP had already implemented the specialist’s recommendations in primary care: “She didn’t say eConsult at that time, she just said ‘a referral.’” **(Patient #5).**

#### Patient concern about cost

Some patients associated the need for a specialty consultation with out-of-pocket cost, and didn’t differentiate between the cost of an in-person specialty visit and the perceived cost of an eConsult. A PCP stated, “Sometimes they [patients] get the gist of it but don’t really put all the pieces together… once you start talking about, you have to do a consult, they start thinking about the money it will take, and then they sometimes aren’t listening to everything until you say it’s free.” **(PCP #9).**

#### Preference for in-person specialty care

Some patients who viewed their condition as severe or complex preferred an in person specialist visit over an eConsult. A patient who had received a Gastroenterology eConsult explained: “When it’s a minor problem, [eConsults are] a good thing, because due to the lack of doctors, I don’t think they have as much time for so many sick people. If there’s a way they can embrace [eConsults] a little more, that’s fine with me…[but] with a major disease, I think that then it’s necessary to personally go to a specialist.” **(Patient #3).** PCPs also described some situations when it was more appropriate for patients to see a specialist face-to-face than to receive an eConsult: “Some of my patients that need referral actually need hands-on, face-to-face consult. So I wouldn’t use an eConsult for that purpose. Like I had a patient yesterday who had a swollen ankle, and she’s uninsured, I wasn’t gonna put in an eConsult, I [would] rather send her to like an ortho[pedics] urgent care clinic, you know.” **(PCP #6).**

#### Limitations of the model

PCPs discussed the difficulty of presenting a patient case when the eConsult specialist could not physically assess the patient. A PCP noted, “In healthcare, we put our hands on people… It’s difficult to be a third-party assessor and to be getting secondhand information. Even though I’m giving them as much information as I can, I sometimes don’t know the right questions to ask… you wanna feel those knuckles, you wanna listen to that heart. You know, you wanna have that physical assessment. I think all practitioners do.” **(PCP #2).** Another expressed that some eConsult specialties required that the request include additional information about the patient: “The least [used are] probably Ortho[pedics] and Derm[atology], just because I think it’s a little bit more difficult to do all the things that possibly they would need in order to give us a proper consultation.” **(PCP #7).**

#### Workflow issues

A PCP described their enthusiasm to submit an eConsult and inability to prepare the case when a tool they needed didn’t work as expected: “I haven’t had access to use [Dermatology eConsults] because my [dermatology imaging] camera app isn’t working so well…I’ve done Derm[atology]…because I love to cut that stuff out myself.” **(PCP #3).**

Some PCPs also raised concerns about time and efficiency. A PCP described receiving an eConsult for a patient they felt needed a face-to-face encounter: “I send a referral [for an uninsured patient] and it automatically does an eConsult even though I know that they need a face-to-face. I think it’s a little waste of time between myself and whoever the eConsult doctor is reviewing the records ‘cause it’s like, ‘Good workup; they need a face to face appointment.’” **(PCP #5).** A second PCP described multiple rounds of communication between PCP and specialist as a potential drawback: “Sometimes the back and forth, or having to ask another question gets a little tricky… [The Referral Coordinator] thankfully is very on top of all these things, and then usually follows through if there’s anything that we’re missing, or if we haven’t responded to something, or if we haven’t seen a result.” **(PCP #9).**

## Discussion

We describe an in-depth exploration of patient and PCP views and opinions regarding uninsured patients’ experience seeking specialty care and the use of a mature, well-utilized eConsult system intended to help improve specialty care access for uninsured patients at a large, multisite FQHC in central Texas. Comments from providers and their uninsured patients underscore the access barriers uninsured patients face when seeking specialty care, including high out-of-pocket cost, lack of specialists willing to see uninsured patients, travel time, distance, and difficulty, and the role of health policy legislation in limiting patients’ ability to afford insurance. Previous studies have documented substantial cost savings to insurance payers with eConsult, [21, 22, 35] and potential savings to patients from reduced out-of-pocket expense, travel costs, or missed work [23, 36, 37]. Both PCP and patient comments highlighted the centrality of cost as a major barrier to receiving needed care, and the benefit of eConsults in reducing or avoiding many of those costs. Comments also highlighted the often unrecognized negative impact of stigma on uninsured patients seeking specialty care. Stigma against uninsured patients and those of low socioeconomic status is well-documented as a contributor to negative patient experience and negative health outcomes [38–40].

Efforts are underway across the country to align payment models to expand the scope and improve the quality of primary care. The importance of primary care and the value of the relationship between patients and their trusted PCP was evident in many of the patients’ comments. eConsults offer an important tool to strengthen that relationship by expanding the scope of primary care, educating PCPs, and allowing more patient care to be provided within their practices. FQHCs are patient- and community-focused by design and place heavy emphasis on addressing the complex medical, behavioral, and social needs of low-income, marginalized populations. These practices tend to be located in the communities where such patients live, and go a long way towards meeting patients’ needs in a patient-centered and holistic manner. Although not all patients inherently understood that eConsults helped them receive more care in their primary care practice, most patients seemed to appreciate the general concept. Patients who expressed attachment to and trust in their primary care provider were particularly supportive of receiving an eConsult as opposed to visiting a specialist.

Our findings also suggest that the concept of an eConsult may not be intuitive to patients. Many struggled to understand the process and seemed to expect that communication between different medical providers was simply how health care delivery took place. These comments are consistent with those described in previous work where patients expressed the importance of clear communication around requesting a specialty eConsult and communicating the results, with some also expressing interest in being involved in the decision to request an eConsult and in having access to the specialist’s verbatim response [29]. Increased emphasis on patient-provider communication, provider training, and shared decision making between patient and provider can help to ensure that patients understand and are empowered to participate in their care decisions [41].

The U.S. Department of Health and Human Services’ Healthy People 2030 plan includes a national objective focused on improving health care access and quality [42]. In the U.S., an estimated 98 million people live in Health Professional Shortage Areas (HPSAs), 15 million live in 3400 + Medically Underserved Areas (MUAs), and 5 million are members of HRSA-designated Medically Underserved Populations (MUPs) [43, 44]. Telehealth and digital health platforms are increasingly seen as crucial to providing equitable, patient-focused access to health services [45, 46]. Technology-enhanced care tools, including eConsults, have the potential to diminish access and health outcome disparities and bolster capacity to adapt care to patients’ needs [7, 47–49]. These study findings highlight and provide context regarding the extent of the challenge posed by limited access to specialty care for patients without insurance, including those in rural locations.

This study provides new information and a broader understanding about PCPs’ experience using eConsults as part of routine primary care for uninsured patients. Our findings suggest that PCPs fully recognized the benefit of eConsults for their uninsured patients, and saw the educational value that they provided. Overall, the PCPs we interviewed did not perceive the eConsult process as burdensome or requiring too much additional work. As the eConsult workflow mirrored the workflow for requesting an in-person specialty referral, their descriptions of eConsult workflow challenges largely centered on ensuring that the specialist had the necessary information to return an actionable recommendation. This contrasts with prior research findings that some PCPs are concerned about eConsults creating more work for them [50]. However, the preponderance of comments suggest that PCPs and patients largely viewed eConsults as a beneficial tool for addressing challenges in specialty care access due to financial and/or geographic barriers. Evidence suggests that eConsults have benefit beyond helping to meet the needs of uninsured and/or rural-dwelling patients. However, further studies should examine whether populations with fewer barriers to access accept eConsults as an alternative for expedient specialty care access versus face-to-face specialty visits, and whether PCPs view eConsults as a useful tool for such patients.

Strengths of this study include its pragmatic, ecological approach to capturing information about all facets of a healthcare service from the care team members who requested it and the patients who utilized it. Our study evaluated real-world implementation of a novel eConsult program, and captured frontline user perspectives.

This study is limited by its focus on a single multi-site FQHC and subsets of PCPs who requested eConsults and patients who received them. While the focus was on eConsults for uninsured patients, PCP comments suggest that they also used eConsults more broadly for insured patients with other limitations to access. Although patients’ recollection of the visit where they received an eConsult may have diminished over time, interviews also captured patients’ perspectives on general use of eConsults for people served at FQHCs and provided context about the patient’s lived experience receiving care while uninsured, and their thoughts about eConsults as a healthcare modality.

## Conclusions

Improving access to care for uninsured patients is essential to reduce health disparities. This study demonstrates that patients and PCPs are willing to embrace eConsults in order to surmount gaps in specialty care. These findings may help inform decisions about how to adopt new tools such as eConsults to further strengthen primary care, improve patient outcomes, and lower costs.

### Electronic supplementary material

Below is the link to the electronic supplementary material.

Supplementary Material 1

## Data Availability

The datasets used and/or analysed during the current study are available from the corresponding author on reasonable request.

## Declarations

### Authors’ information

DA is a primary care physician and is VP and Chief Quality Officer at Community Health Center, Inc., a primary care federally qualified health center which began building its own electronic consultation (eConsult) program in 2010 to expand access to specialty care for its marginalized, uninsured and underinsured patients. That program led to the creation of a nationwide, not-for-profit eConsult provider now known as ConferMED (incorporated in 2015), which now supports FQHCs and other safety-net practices in 26 states. In addition to his role as President at ConferMED, he is Director Emeritus of Weitzman Institute, a research, evaluation, education, and policy organization, where he currently serves as a Senior Research Scientist. LB, LG, and IV are members of the research team at Weitzman Institute, which works to ensure that effective, efficient, and equitable care is available to all. At the time this work was conducted, TA was Chief Medical Information Officer at Lone Star Circle of Care, a large federally qualified health center with > 55 clinics in two dozen hub locations spanning 6 counties in central/southeast Texas. She is now Chief Medical Officer at Care Message, a 501(c)(3) nonprofit committed to helping underserved populations improve their access to healthcare using mobile technology.

## Abbreviations

eConsults: Electronic consultations
FQHC: Federally qualified health center
M: Mean
PCP: Primary care provider
SD: Standard deviation
SRQR: Standards for reporting qualitative research
