# Supplementary material for: A qualitative investigation of uninsured patient and primary care provider perspectives on specialty care eConsults
Source: BMC Health Serv Res. 2023 Oct 20;23:1133. doi: 10.1186/s12913-023-10086-6 (PMC10589958; doi:10.1186/s12913-023-10086-6)
Supplement: Supplementary file 1 — Supplementary Material 1 [file 12913_2023_10086_MOESM1_ESM.docx]

**Appendix 1: Patient and PCP Semi-Structured Interview Guides**

**Patient Interview Guide**

*Prior to beginning the interview, each patient was provided with a brief verbal description of the care they had received. The interviewer then used the following questions to guide the interview, substituting patient-specific information for each [SPECIALTY CONDITION] placeholder.*

List of eConsult specialties:

Allergy, Cardiology, Dermatology, Endocrinology, ENT (Otolaryngology), Gastroenterology, Hematology, Nephrology, Neurology, Orthopedics, Pain Medicine, Pulmonology, Rheumatology, Urology

**Access to specialty care among uninsured, rural patients in Texas:**

1. Specialists are doctors who have additional training to care for complex conditions and specific parts of the body. People will sometimes see a doctor who sees patients specifically for [SPECIALTY CONDITION] for their care, in addition to their primary care provider.
   - Do you know why your doctor recommended that you see a specialist for [SPECIALTY CONDITION]? If so, please tell us about the reason.
2. Tell us about a time when you needed to see a specialist doctor other than your primary care provider for [SPECIALTY CONDITION].
   - What was it like for you to make the appointment?
   - What was it like for you to visit the office or receive care by telehealth (video or phone)?
   - What was it like for you to pay for the visit?
3. Would you prefer to get care for [SPECIALTY CONDITION] from your doctor at [FQHC] if you could? Why or why not?
   - What are the benefits of receiving care for [SPECIALTY CONDITION] from your doctor at [FQHC] instead of from a different doctor who specializes in that condition?
   - What are the benefits of receiving care for [SPECIALTY CONDITION] from someone other than your doctor at [FQHC], like a specialist who treats patients with [SPECIALTY CONDITION]?
   - In your opinion, what would be the best way for you to get care for [SPECIALTY CONDITION]?

**Awareness and Opinions of eConsults:**

**General Awareness of eConsults**

eConsults allow your doctor to send your medical information electronically to a specialist who can review it and send back their recommendations. Many times this means that you don’t need to physically go to see the specialist, or see them by video or phone.

1. Has your provider ever discussed eConsults with you? What did they say?
2. Did they explain to you what eConsults were?

**Awareness of eConsults Used as Part of Patient’s Own Care**

When you saw your doctor on [DATE], you discussed [SPECIALTY CONDITION] with them. They used an eConsult to obtain a specific recommendation about you and your care from a doctor who specializes in [SPECIALTY CONDITION].

1. Did your doctor mention to you that you had received an eConsult?
2. Did they review the eConsult recommendations from the specialist with you?
3. What do you think about this service (the eConsult)?
   - Do you think the advice your doctor gave you about [SPECIALTY CONDITION] was helpful for you?
   - Do you think it helps your doctor to use an eConsult with a doctor who specializes in [SPECIALTY CONDITION]?
4. Do you think it is helpful to patients that doctors can ask for an eConsult on [SPECIALTY CONDITION] from a doctor who specializes in it?

**PCP Interview Guide**

**General Perceptions of and Experiences with eConsults:**

1. Please describe in your own words what an eConsult is.
   - What are the benefits of obtaining an eConsult for patients in your panel?
   - What are the drawbacks?
   - In general, how often do you use eConsults?
   - Are there certain specialties that you use more often? Less often? Why?
   - Are there certain types of patients that you use eConsults for more\less often? Why?
2. Tell us what it is like to discuss an eConsult recommendation with a patient during a primary care visit.
   - Do you talk about eConsults with patients when you order them?
     - Do patients understand what an eConsult is?
     - How do patients feel when you order an eConsult for them?
   - If you do not tell patients that they have received an eConsult, why not?
3. What specialties have the greatest demand for an eConsult at your practice site, or in your city or geographic area? At [FQHC organization] in general?
4. What structural, policy, legal, or regulatory barriers are you aware of in Texas that impact patients’ access to specialty care?
   - For example, economic barriers like insurance or payment, difficulty accessing care or treatment, fear or concern about stigma or discrimination?

**A Patient’s View of an eConsult:**

*We would like to discuss a patient in your panel who received a/an [SPECIALTY NAME] eConsult from you.* ***This patient has provided us with their permission to share their responses with you.***

Please pull up their chart in the electronic health record for your own reference during this part of the interview.

1. Please describe the reason you requested an eConsult for this patient.
2. How did you use the information in the eConsult?
3. What was the impact of using an eConsult on this patient’s care outcomes?
4. *[Interviewer provides a recap of the patient’s responses to questions about receiving an eConsult as part of their care].* Do these comments align with your expectations? Is there anything surprising?
5. If you were seeing a similar patient for this condition today, would you request an eConsult?
    Why or why not?
6. What would an ideal solution for delivering specialty care to patients look like?
   PROBE: Is there another alternative besides referral to specialist or an eConsult?

**WRAP UP:** Is there anything else you would like to share about your experience using eConsults in general, or for this specific patient?
